# Oligomalt, a New Slowly Digestible Carbohydrate, Reduces Post-Prandial Glucose and Insulin Trajectories Compared to Maltodextrin across Different Population Characteristics: Double-Blind Randomized Controlled Trials in Healthy Individuals, People with Obesity, and People with Type 2 Diabetes

**DOI:** 10.3390/metabo14080410

**Published:** 2024-07-26

**Authors:** Odd Erik Johansen, Joel Neutel, Sanjay Gupta, Barbara Mariani, Gerhard Ufheil, Emilie Perrin, Andreas Rytz, Anirban Lahiry, Frederik Delodder, Jaclyn Lerea-Antes, Naomi Ocampo, Maximilian von Eynatten

**Affiliations:** 1 Nestlé Health Science, 1000 Lausanne, Switzerland; 2 Orange County Research Center, Tustin, CA 92780, USA; 3 Nestlé Product Technology Center NHS, Société des Produits Nestlé S.A., Bridgewater, NJ 08807, USA; 4 Nestlé Research and Development Konolfingen, Société des Produits Nestlé S.A., 3510 Konolfingen, Switzerland; 5 SOCAR Research SA, 1260 Nyon, Switzerland; 6 Nestlé Research, Clinical Research Unit, 1000 Lausanne, Switzerland; 7 Nestlé Health Science, Bridgewater, NJ 08807, USA

**Keywords:** clinical trial, dietary carbohydrate, food, digestion

## Abstract

We assessed the glucometabolic effects of oligomalt, a novel fully slowly digestible carbohydrate, compared with maltodextrin, in cross-over randomized controlled trials (NCT05058144; NCT05963594) involving healthy volunteers (HV), people with overweight or obesity (PwO), and people with type 2 diabetes (T2D). We tested 33 g and/or 50 g of oligomalt/maltodextrin, which were dissolved in 300 mL of water and consumed after fasting in the morning. The primary exploratory endpoint was the incremental area under the curve (iAUC) for postprandial glucose, assessed by frequent blood sampling over 3 h. Insulin levels were also assessed. In the HV cohort, a 4 h hydrogen breath test was performed with 15 g of inulin as a positive control. Analysis was performed by a mixed model. Oligomalt elicited a lower post-prandial glucose response compared to maltodextrin in HV (50 g, n = 15 [7 women], mean age/BMI 31 years/22.6 kg/m^2^), in PwO (33 g and 50 g, n = 26 [10 women], age/BMI 44 years/29.9 kg/m^2^, mean HbA1c 5.3%), and in people with T2D (50 g, n = 22 [13 women], age/BMI 61 years/31.8 kg/m^2^, HbA1c 7.4%), with significant reductions observed in PwO and T2D for the 0–1 h window (HV: −19% [*p* = 0.149]/PwO_33g_-38% [*p* = 0.0002]/PwO_50g_-28% [*p* = 0.0027]/T2D-38% [*p* < 0.0001]; the 0–2 h window (HV: −17% [*p* = 0.311]/PwO_33g_-34% [*p* = 0.0057]/PwO_50g_-21% [*p* = 0.0415]/T2D-37% [*p* < 0.0001]), and the 0–3 h window (HV: −15% [*p* = 0.386]/PwO_33g_-30% [*p* = 0.0213]/PwO_50g0_−19% [*p* = 0.0686]/T2D−37% [*p* = 0.0001]). The post-prandial insulin response was significantly lower, by 38–60%, across all populations, dose, and time points, with oligomalt. In HV, the breath-hydrogen pattern was comparable between oligomalt and maltodextrin, but increased significantly with inulin. These data support the glucometabolic advantages of oligomalt over maltodextrin, hence confirming it as a healthier carbohydrate, and underscoring its full digestibility. This therefore opens up the possibility for the incorporation of oligomalt in relevant food products/matrices.

## 1. Introduction

Carbohydrates in the diet are major macronutrients for humans, and many dietary guidelines provide recommendations for daily energy intake that should be obtained from this macronutrient. The Food and Drug Administration (FDA) has, for example, suggested 275 g of carbohydrates when eating a 2000-calorie diet, which may be higher or lower according to individual calorie needs [1], whereas the Dietary Guidelines for Americans recommend that carbohydrates should make up 45–65% of total daily calories of people aged two and older [2]. In some regions of the world, in particular Asia, the percentage consumed in reality can be higher. Under special circumstances, these intakes are significantly higher, like amongst professional athletes and those involved in endurance sport, and some advocate an intake of 5–7 g/kg/day for general training needs, and 7–10 g/kg/day for the increased needs of endurance athletes [3].

According to the World Health Organization (WHO), simple carbohydrates include single sugars (monosaccharides) and disaccharides, oligosaccharides, and polysaccharides [4]. Furthermore, they also provide definitions for “total sugars” that include intrinsic (naturally occurring) sugars, such as sugars in the structure of intact fruit and vegetables, milk sugar (lactose), all added sugars in foods and beverages [4], and “free sugars”, which include monosaccharides and disaccharides added to foods and beverages by the manufacturer, cook, or consumer. In addition, there are sugars that are naturally present in honey, syrups, fruit juices, and fruit juice concentrate. “Added sugars” include sugars added to food during food processing, sugars used as sweeteners, and sugars from honey and concentrated fruit or vegetable juices. Added sugars do not include naturally occurring sugars, such as sugars in the intact cell walls of fruit and vegetables, or sugars present in milk [4]. 

Typically, the distribution of the types of consumed carbohydrate varies, and is approximately 60% in the form of polysaccharides, mainly starch, whereas disaccharides, like sucrose and lactose, contribute around 30–40%, while monosaccharides, like glucose and fructose, are usually consumed in much lower amounts [5]. 

Current US dietary guidelines recommend consuming 10% or less of one’s daily calories from added sugars [2], and the European Food Safety Authority (EFSA) concluded in 2022 that the intake of added and free sugars should be “as low as possible in the context of a nutritionally adequate diet” [6]. Over recent decades (e.g., data from 9 National Health and Nutrition Examination Survey cycles 1999–2016 among adults aged 20 years or older), the percentage of energy intake from low-quality carbohydrates has reduced, with significant increases in the percentage of energy intake from high-quality carbohydrates [7]. However, the average American consumes around 270 calories per day, i.e., ~13% of their daily calories, in this form [2], a number that aligns to a large extent with reports from Europe among adults and the elderly, i.e., 8–14% [8].

Excess ingestion of sugars and carbohydrates that are rapidly digested can lead to a number of unfavorable metabolic alterations, e.g., increasing triglycerides, free fatty acids, and lipids [9,10,11,12], and are associated with several health conditions (e.g., gout, fatty liver, obesity, type 2 diabetes mellitus [T2D], and cardiovascular disease). The WHO recommends an upper limit of free sugars at 10% of calories, with an ultimate goal of reducing sugar consumption to 5% of calories [13], and the American Heart Association Nutrition Committee recommends women and men consume no more than 100 and 150 kcal of added sugar per day, respectively [14].

A growing focus in the health and food industry is to replace added sugars and rapidly digested carbohydrates in food matrices, with slowly digestible carbohydrates (SDC), i.e., carbohydrates that are completely digested in the small intestine but at a slower rate [15]. Since SDC are fully but slowly digested throughout the small intestine due to their structural complexity, their net digestion profiles typically result in a less pronounced glycemic response coupled with a lower insulin requirement [16]. Oligomalt is a novel SDC not yet incorporated into commercial products, and has been previously studied in healthy volunteers (HV) where it was seen that a single consumption of a dose of 33 g, compared with 33 g of maltodextrin, led to a significantly lower glycemic excursion over 3 h [17], and when studied over a period of 7 days, with consumption doses of up to 180 g per day (provided over four intakes of 45 g), in addition to a standard diet, was well tolerated compared with similar amounts of maltodextrin [18].

Herein we describe the glucometabolic effects of oligomalt when assessed in cross-over randomized controlled trials across different populations, including HV, people with overweight or with obesity (PwO), and people with T2D. We also evaluated its digestibility by a hydrogen breath test compared to a positive control (fiber) in the HV cohort.

## 2. Materials and Methods

### 2.1. Study Design

Three randomized–controlled, double-masked, cross-over studies were conducted and involved three different populations of both genders: HV, PwO, and people with T2D. Participants consumed a single serving of either oligomalt or maltodextrin with a wash-out period of approximately one week between the different product intakes. The doses investigated were 50 g of maltodextrin/oligomalt and 15 g of inulin (as a non-digestible control) in the HV cohort, and followed a triple cross-over design. In the T2D cohort, the dose investigated was 50 g of maltodextrin/oligomalt and it followed a traditional cross-over design, whereas the cohort of PwO consumed both 33 g and 50 g of each product in a quadruple cross-over design. The additional testing of a 33 g dose in PwO allowed for an assessment of dose–response, and a historic comparison versus a previous study employing 33 g in HV [17].

### 2.2. Study Participants

In brief, key criteria of the HV cohort included the age range of 18–45 years, BMI 18.5–29.9 kg/m^2^, and a requirement to be generally healthy. For the PwO cohort, age had to be ≥18 years and BMI ≥ 25 kg/m^2^ (with a protocol specification to recruit a minimum of 10 and a maximum of 14 people with BMI 25–30 kg/m^2^, or BMI ≥ 30 kg/m^2^, respectively, of the planned minimum 24 participants). For the T2D cohort, the age had to be ≥18 years and, in addition to a diagnosis of T2D, HbA1c had to be 6.5–10.0%. Owing to frequent blood sampling, hematocrit was ≥ 34/40% for females/males, and hemoglobin was ≥ 11.0/13.5 g/dL for females/males, respectively. There was no predefined target for the number of people to be recruited based on race or ethnicity across the cohorts. The full list of inclusion and exclusion criteria is provided in Supplementary Tables S1 and S2.

All participants provided written informed consent, and the experiments were carried out in compliance with the Harmonized Tripartite Guideline for Good Clinical Practice from the International Conference on Harmonisation [19] and the Declaration of Helsinki [20], and conducted at two different study sites. The HV cohort was studied at the clinical research unit at Nestlé Research, Lausanne, Switzerland, and the cohorts involving PwO and people with T2D was studied at the Orange County Research Center, Tustin, CA, USA.

### 2.3. Investigational Products

The maltodextrin used in these studies was “Glucidex^®^ 40” (Roquette Frères, Lestrem, France), a high-dextrose equivalent (DE) maltodextrin (also called glucose syrup; herein we use the term “maltodextrin”) and widely used as a carbohydrate source and/or bulking agent in a variety of food products, mainly constituted by maltooligosaccharides (predominantly α-D-Glc*p*(1→4)-α-D-Glc*p*) with an average DP of 3 and a DE equal to 40. This comparator product, which was isocaloric with oligomalt, was also chosen to allow comparability with previously conducted studies [17,18].

Oligomalt, the novel SDC, is a α-glucan polymer [α-D-Glc*p*-(1↔3)-α-D-Glc*p*], [α-D-Glc*p*-(1↔6)-α-D-Glc*p*], and was produced in collaboration with Evoxx technologies GmBH (Monheim am Rhein, Germany), as previously described in [17]. In short, oligomalt is produced via a transglycosylation reaction catalyzed by the enzyme alternansucrase, with sucrose as a donor of one glucose moiety and maltose as the acceptor substrate. This reaction results in a linear oligosaccharide with alternating α1-6 and α1-3 linkages.

The oligomalt used in this study was comprised of small quantities of free sugars (<5.5% sugars, of which <0.2% was fructose and 5.3% was leucrose, owing to the production steps, but zero glucose, sucrose, isomaltose, or maltose), with an average ± standard deviation DP of 16.5± 0.1 and molecular weight of 2.7± 0.02 kDa. The structure of oligomalt is provided in the Supplementary Figure S1.

In the HV study only, 15 g of inulin powder was consumed (provided by Cosucra—Groupe Warcoing S.A., Warcoing, Belgium, Lot: 2018486975). A dose of 15 g was selected to facilitate an adequate H_2_ response. The dry matter of the powder was 96.3% with inulin representing 90.8% and free fractions of fructose, glucose, and sucrose 9.1%.

### 2.4. Study Procedures

All test products were provided at the respective clinics in the morning of the study visit after an overnight fast (i.e., at least 10 h). They were dissolved in 300 mL of water, and consumed within 10 min. Dedicated personnel, who were not directly involved in the trial, prepared the drinks and provided the products to trial personnel who distributed them to participants. The participants and the trial personnel that administered the products to participants were blind to the product identities.

All participants for each sequence had, thereafter, frequent blood sampling conducted for glucose (all cohorts), insulin (all cohorts), GLP-1 (PwO and T2D cohorts), and PYY (PwO and T2D), with a sampling scheme at t = 0 (just before consumption of the product), and thereafter at the following post-ingestion timepoints t = 15, 30, 45, 60, 90, 120, 150, and 180 min. All blood samples were taken from subjects by venipuncture or cannulation, and serum and plasma were prepared using standard procedures. The study scheme is detailed in Supplementary Figure S2.

A differential number of visits across the groups were employed to adapt the study burden according to baseline conditions. As such, for the group with most comorbidities, i.e., people with T2D, we only tested one dose of oligomalt (50 g) and maltodextrin (50 g), hence requiring only two study visits. For the cohorts that were healthier, slightly more study procedures were deemed acceptable and, as such, for the PwO cohort we tested 33 g and 50 g of both ingredients, requiring four visits. In the HV cohort, we also implemented the H_2_ breath test, which lasted 4 h, and in this cohort we tested 50 g of oligomalt, 50 g of maltodextrin, and 15 g of inulin.

### 2.5. Primary Exploratory Endpoint

The exploratory study hypothesis was that the consumption of oligomalt, compared to maltodextrin, consumed in the morning after fasting, would trigger a lower post-prandial (PP) blood glucose excursion across all study cohorts. Thus, the primary exploratory endpoint was to assess the effects on PP glucose (iAUC) over the full observation period, which included assessments at t = 0 (i.e., pre-consumption assessment), and thereafter until t = 180 min, and we pre-specified to assess during the following windows: 0–60 min, 0–120 min, and 0–180 min.

Glucose was analyzed in EDTA-plasma (Cobas c501 Systems, Roche Diagnostics, Indianapolis, IN, USA).

### 2.6. Other Metabolic Endpoints

Interval blood samples for insulin (serum, Immulite 2000 Analyzer, Consolidated Medical Bio-Analysis, Inc., Cypress, CA, USA) for all cohorts were pre-specified to be assessed during iAUC windows of 0–60 min, 0–120 min, and 0–180 min. For total GLP-1 (EDTA-plasma, MSD, Gaithersburg, MD, USA) and total PYY (EDTA-plasma, Millipore, St. Louis, MO, USA) in the PwO and T2D cohorts, we post-hoc defined the analytical iAUC windows to be 0–90 min and 90–180 min.

Based on the interval blood samples, we also compared the maximum concentration (Cmax) of glucose and insulin and the time it took to reach the Cmax (i.e., Tmax).

Additionally, for glucose in all cohorts, we assessed the glucose variability, expressed as interquartile range [21], % of people with mild hypoglycemia during the observation, defined as a glucose value ≤ 70 mg/dL, a threshold for neuroendocrine responses to falling glucose in people without diabetes [22], and glucose stability expressed as the slope of glucose decline from Cmax and until 180 min. Additional insulin analysis was through the calculation of the insulin index (II) [23], a measure of the insulin response to various ingredients or foods relative to the insulin response to a comparator (here maltodextrin), which was assigned a score of 100.

### 2.7. Breath Hydrogen Test

Hydrogen (H_2_) breath testing is used for the detection of carbohydrate malabsorption [24,25]. The principle relies on the detection of hydrogen in the exhaled air resulting from bacterial fermentation of carbohydrates that are not absorbed, mainly in the colon, and a rise in hydrogen of ≥20 parts per million (ppm) from the baseline during a breath test is considered positive for maldigestion [24]. Here, the H_2_ breath test was performed only in the HV cohort to assess carbohydrate digestibility for 4 h, with measurements at t = 0 (just before consumption of oligomalt 50 g, maltodextrin 50 g, or inulin 15 g) and thereafter at t = 30, t = 60, t = 90, t = 120, t = 150, t = 180, t = 210, and t = 240 min (Supplementary Figure S2). Breath samples were analyzed with a breath analyzer (Lactotest 202, M.E.C., Fosses-la-Ville, Belgium).

The breath hydrogen content was also used as a parameter to assess the estimated fiber content (EFC) of oligomalt. This was enabled by giving a positive control, i.e., a known amount of fiber (inulin), which thereby provided an estimate of the amount of carbohydrate (starch and fiber) not absorbed for oligomalt [26].

### 2.8. Safety and Adverse Events (AEs)

General safety laboratory tests were performed at the screening visit. Occurrence of AEs were proactively assessed by queries at all post-screening visits, and all AEs (spontaneously reported or enquired, as well as those observed) during the course of the study were captured and summarized descriptively. 

AEs were coded using the Medical Dictionary for Regulatory Activities (MedDRA), Version 23.0.

### 2.9. Statistical Methods and Sample Size Considerations

Sample size estimations for these exploratory studies were made separately for the HV cohort and the cohorts with metabolic compromise (i.e., PwO and people with T2D). For the HV cohort, we based the assumption of the effect on glucose from a previous study investigating 33 g oligomalt/maltodextrin in 16 healthy subjects, using a continuous glucose monitoring device, where the observed reduction for the 3h-iAUC was 33%, with a standard deviation of 45% [17]. Assuming similar relative magnitude of effect and variability in this new study, a sample size of n = 15 would be required (alpha = 5%, two-sided, power = 80%). Allowing for one potential drop-out, our target enrolment group was n = 16.

For the cohort involving PwO and people with T2D, we did assume a higher variability, hence assuming that this would lead to a slightly higher number required, with the largest sample size needed in the PwO group owing to the additional trial complexity (quadruple cross-over instead of double-cross over) that increased the likelihood for premature discontinuation. Minimum target enrolment for the PwO cohort was set at n = 24 and for the T2D cohort at n = 20.

### 2.10. Analysis

Patient characteristics were described using mean (standard deviation) for continuous variables and proportions for categorical variables. Descriptive data for graphical presentation was shown as mean (standard error). The differences in endpoints between oligomalt and maltodextrin in PwO and in people with T2D, and between oligomalt, maltodextrin, and inulin in the HV groups, were assessed by comparing changes in iAUC between the interventions over different time periods using ANOVA (linear mixed model with product, period, and sequence as fixed effects and participants as random effects nested within sequence) and expressed as effect estimates (95% CI) and relative differences between treatment groups (calculated in % as estimated treatment difference/estimated mean for comparator × 100%).

The model was adjusted for investigational product and period. *p*-values were not adjusted for multiple comparisons. In the event that the model ANOVA did not converge, a log-transformation was performed and rate ratios calculated.

We also assessed if there were differences in both maximum levels of the biomarkers reached (Cmax) and time to reach maximum concentration levels of the biomarkers (Tmax). IQR was calculated as IQR = Q3 (measured glucose values over 3 h) − Q1 (measured glucose values over 3 h), and insulin index was calculated as 2h-iAUC_Ins_ (oligomalt)/2h-iAUC_Ins_ (maltodextrin) × 100.

The analysis of H_2_ in breath was conducted in the HV population and compared to differences in iAUC (log-transformed) at timepoints 0–3 h and 0–4 h. The EFC in % was calculated as: EFC = $\frac{oligomalt-maltodextrin}{inulin-maltodextrin}\times \frac{15g}{50g}\times \left(100\%\right)$.

## 3. Results

The study involving the HV cohort was conducted in Oct–Nov 2021, and the study involving PwO and people with T2D in Jan–March 2023.

In total, 16 participants were screened in the HV cohort, of whom 15 were enrolled and randomized (representing the analysis population for efficacy and safety), and all completed their allocated sequence and test periods. In the cohort of PwO, in total 33 participants were screened, of whom 7 were screen failures, leading to 26 participants enrolled and randomized (representing the analysis population for efficacy and safety), with 24 completing their allocated sequence and test periods (one participant withdrew consent after the first period, and one after the second period). In the cohort of people with T2D, 37 participants were screened and 15 were screen failures, providing 22 participants that were enrolled and randomized (representing the analysis population for efficacy and safety), of whom 20 completed their allocated sequence and test periods (two participants withdrew their consent after the first period). The consort diagram is presented in Supplementary Figure S3.

As expected, the mean age of the participants in the different cohorts varied (Table 1), where the HV was youngest at mean age 31 years, and the PwO and T2D cohorts 44 and 61 years, respectively. Both males and females were well represented. Most participants were white (100% in the HV cohort, 54% in the PwO cohort, and 45% in the T2D cohort), but the study cohorts of PwO and people with T2D, respectively, also had a representation of Asians (respectively, 4% and 23%), and Black or African-Americans (respectively, 35% and 32%). Mean BMI was lowest in the HV cohort (22.6 kg/m^2^), and this was 29.9 kg/m^2^ and 31.8 kg/m^2^ in the PwO and T2D cohorts, respectively. The study successfully enrolled a balanced number of participants with BMI < 30 vs. ≥30 in the PwO cohort (Supplementary Table S3), and there were 13 subjects in each category. Those with BMI 25–30 were slightly younger (mean age 40) and had a mean BMI of 27.2 kg/m^2^, and the group with BMI ≥ 30 had a mean age of 47 and a mean BMI of 32.7 kg/m^2^.

Use of concomitant medications was frequent in the T2D cohort with, e.g., 82% taking metformin, 46% taking RAAS medications, and 46% taking lipid-lowering medications. Only one individual in the PwO used concomitant medication, while none in the HV cohort did.

### 3.1. Post-Prandial Glucose Trajectories

**Figure 1:**
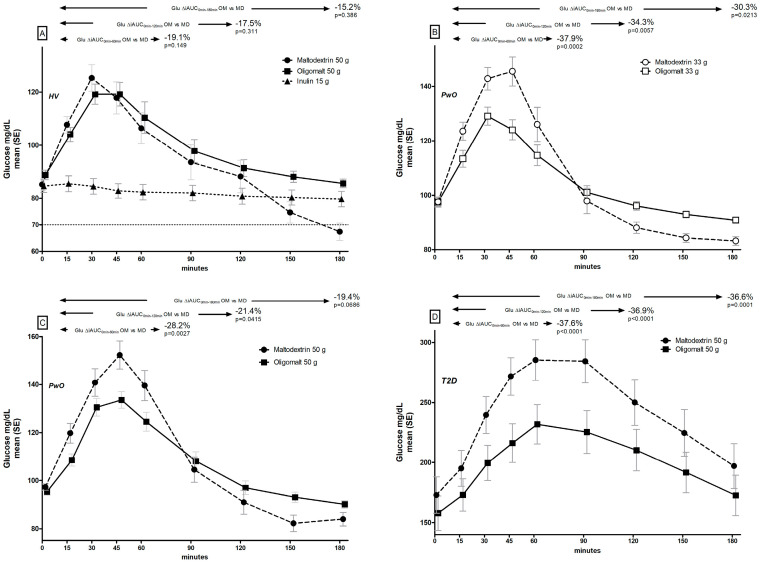
Post-prandial glucose (glu) trajectories by consumption of maltodextrin (MD), oligomalt (OM), or inulin in HV (**A**), PwO (**B**,**C**), or people with T2D (**D**).

There were distinct differences in glucose trajectories following consumption of the various ingredients (Figure 1, Table 2). In the HV cohort, there was a 15–19% lower iAUC across all time points assessed (0–60 min, 0–120 min, and 0–180 min) when comparing 50 g oligomalt with 50 g maltodextrin (Figure 1A), which did not reach statistical significance. As expected, inulin had no notable effect on glucose. The maximum increase in glucose concentration was significantly lower for oligomalt compared to maltodextrin in the HV cohort (−10.8 mg/dL, *p* = 0.032), whereas there was no significant difference in time to maximum glucose peak (oligomalt: 40 min; maltodextrin: 34 min, *p* = 0.249).

In the cohort of PwO, significantly lower iAUC (0–60 min, 0–120 min, and 0–180 min) were seen for both the 33 g (by 30–38%; Figure 1B) and the 50 g dose (by 19–28%; Figure 1C). The differences in maximum increase in glucose were also significantly lower for oligomalt (Table 2), regardless of dose, whereas time to maximum peak glucose results were similar (33 g oligomalt: 39 min; 33 g maltodextrin: 39 min, *p* = 0.865; 50 g oligomalt: 44 min; 50 g maltodextrin: 46 min, *p* = 0.816). No notable differences in these patterns across subgroups with BMI 25–30 kg/m^2^ or BMI ≥ 30 kg/m^2^ (Supplementary Figure S4).

Figure 1D depicts the glucose trajectories in the T2D cohort with a 50 g dose, and in addition to the higher baseline glucose values (with a small between-group difference at t = 0 for maltodextrin at 158 mg/dL vs. oligomalt at 173 mg/dL), the shape of the glucose curves are different from those observed in people without T2D (Figure 1A–C), which was not unexpected. However, there was also a significantly lower glucose response evident for oligomalt, where iAUC were 37–38% lower than for maltodextrin. The corresponding incremental peak glucose was also lower (Table 2), but not time-to-peak glucose (50 g oligomalt: 69 min; 50 g maltodextrin: 66 min, *p* = 0.597).

Across all the cohorts, glucose variability was significantly lower for oligomalt compared to maltodextrin, as expressed by IQR (Table 2), and glucose stability was higher, expressed with a less steep slope reduction in glucose following peak value until the end of the observation. The latter is of relevance for the observation that at timepoints 150 min and 180 min, respectively, 40% (n = 6) and 60% (n = 9) of participants in the HV cohort when consuming maltodextrin had glucose values ≤ 70 mg/dL, conventionally defined as mild hypoglycemia, whereas the corresponding proportion for oligmalt was 7% (n = 1) and 0% (n = 0). Owing to the higher baseline values (Figure 1B–D) in PwO and T2D, we observed fewer incidents of mild hypoglycemia in these cohorts. In the T2D there was none, and in PwO with 50 g maltodextrin its occurrence was 28% (n = 7) at t = 150 min, and 8% (n = 2) at t = 180 min, whereas there was none with 50 g oligomalt (at either timepoint). In PwO with the 33 g dose, its occurrence was 4% (n = 1) at 150 min and 4% (n = 1) at 180 min with maltodextrin, and there was none with oligomalt.

### 3.2. Post-Prandial Insulin Trajectories

**Figure 2:**
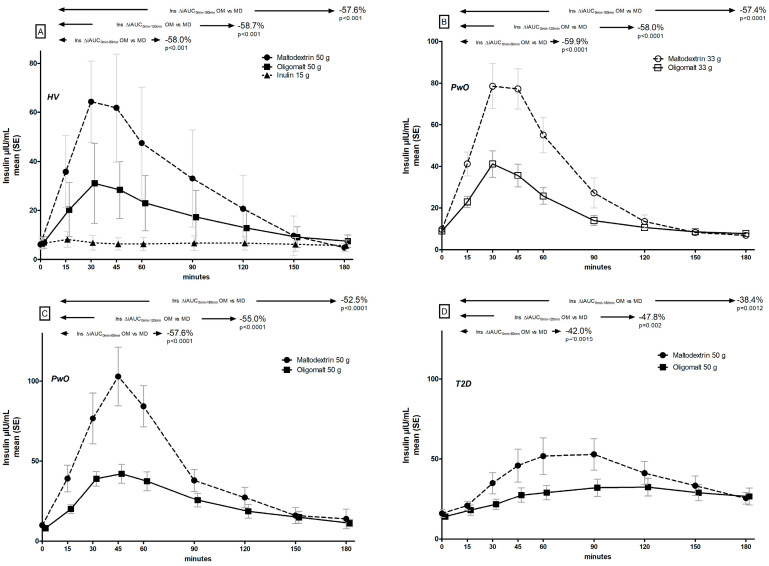
Post-prandial insulin (ins) trajectories by consumption of maltodextrin (MD), oligomalt (OM), or inulin in HV (**A**), PwO (**B**,**C**), or people with T2D (**D**).

As for glucose, there were also distinct differences in insulin trajectories following consumption of the various ingredients (Figure 2, Table 3). In the HV cohort (Figure 2A), there was a 58–59% lower iAUC across all time points assessed (0–60 min, 0–120 min, and 0–180 min) when comparing 50 g oligomalt vs. 50 g maltodextrin (all statistically significantly different); as expected, inulin did not induce a notable insulin response. The maximum increase in insulin concentration was significantly lower with oligomalt vs. maltodextrin, as was the insulin index (Table 3), whereas there was no difference in time to maximum insulin peak.

In the cohort of PwO, significantly lower insulin iAUC (0–60 min, 0–120 min, and 0–180 min) were seen (Figure 2B,C) both for the 33 g (by 57–60%) and for the 50 g dose (by 53–58%). The differences in maximum increase in insulin with these doses were also significantly lower with oligomalt (Table 3), with the largest absolute difference seen with the 50 g dose, and both doses reduced the insulin index. Time to maximum peak insulin was similar (Table 3). There were no notable differences in these patterns between the subgroups with BMI 25–30 kg/m^2^ or BMI ≥ 30 kg/m^2^ (Supplementary Figure S5).

The insulin trajectories in the T2D cohort with 50 g doses (Figure 2D) showed an expectedly flatter curve than in the cohorts without T2D. However, there was also a significant lower insulin excursion (with iAUC being reduced by 38–48%, and lower insulin maximum concentration and lower insulin index) with oligomalt vs. maltodextrin (Table 3).

### 3.3. Post-Prandial GLP-1 and PYY in PwO and People with T2D

**Figure 3:**
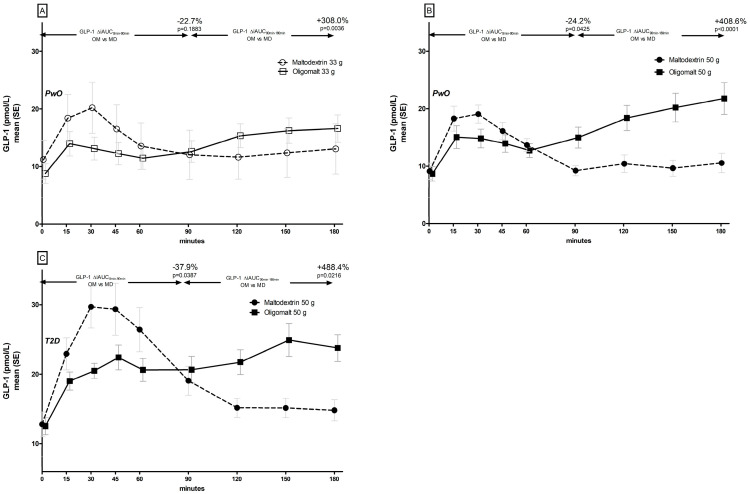
Post-prandial GLP trajectories by consumption of maltodextrin (MD) or oligomalt (OM) in PwO ((**A**): 33 g and (**B**): 50 g)), or people with T2D (C).

**Figure 4:**
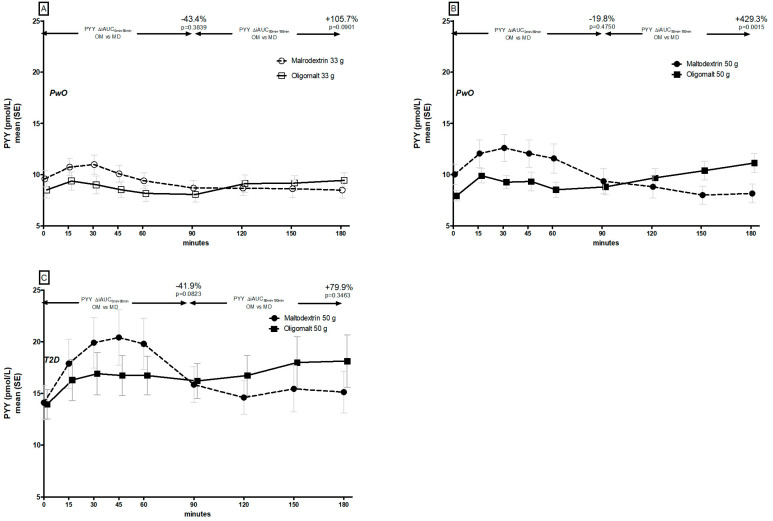
Post-prandial PYY trajectories by consumption of maltodextrin (MD) or oligomalt (OM) in PwO ((**A**): 33 g and (**B**): 50 g), or people with T2D (**C**).

There were distinct differences in the GLP-1 (Figure 3) and PYY (Figure 4) trajectories following consumption of oligomalt and maltodextrin in PwO and in people with T2D.

In PwO (Figure 3A,B), with both doses, the GLP-1 response was lower for the first half (0–90 min) of the observation period with oligomalt, but higher in the last part (90–180 min). This response appeared to be dose-dependent and was consistently observed, regardless of BMI categories (Supplementary Figure S6). In people with T2D, the pattern was similar (Figure 3C), but the maximum concentrations appeared higher than in PwO.

PYY (Figure 4A,B, Supplementary Figure S7) followed a similar pattern as GLP-1 in PwO, albeit with an attenuated response during the 90–180 min period. In people with T2D, the baseline PYY values were higher (Figure 4C), but the pattern was consistent with that of PwO.

### 3.4. Hydrogen Breath Test in HV

**Figure 5:**
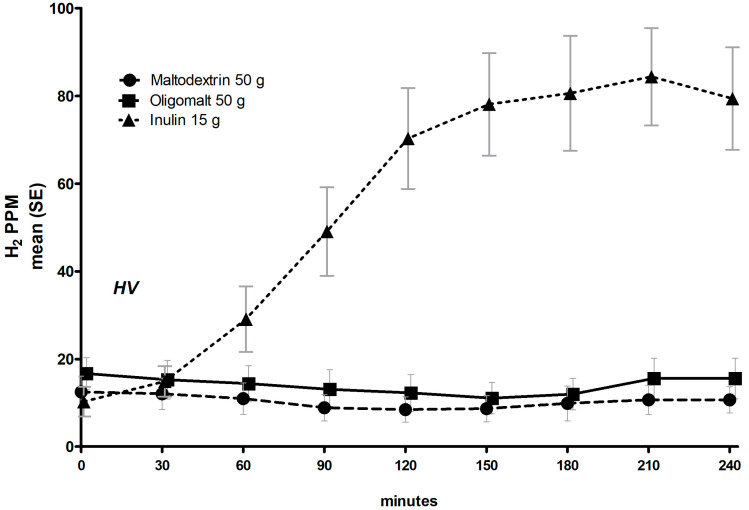
Trajectory of hydrogen content in breath over 4 h of healthy volunteers following consumption of 50 g oligomalt, 50 g maltodextrin or 15 g inulin.

Hydrogen in breath was distinctly different over time (Figure 5) between maltodextrin and inulin, and between oligomalt and inulin, but not between maltodextrin and oligomalt.

Assessing the differences in iAUC statically, there were significant differences for breath hydrogen iAUC for 0–3 h and 0–4 h between each carbohydrate and inulin, but not between the two carbohydrates (Table 4). EFC for oligomalt demonstrated a value <1%.

### 3.5. Adverse Events

One individual in the T2D cohort developed a cough and upper respiratory congestion for 12 days, which were not deemed related to the product intake, and were of mild severity and resolved fully. No adverse events were reported in any of the other cohorts.

## 4. Discussion

These cross-over studies were conducted to expand the understanding of the metabolic effects of oligomalt beyond what had been observed in healthy volunteers (HV) [17,18], i.e., by involving individuals with other demographics, of more advanced ages, of higher BMI, with lower insulin sensitivity and higher insulin resistance, with the presence of comorbidities, and who use concomitant medications for various conditions. The studies demonstrated that oligomalt vs. maltodextrin induces a lower glycemic as well as insulinemic response, regardless of population, and that in PwO a comparatively higher dose of maltodextrin vs. oligomalt elicits a larger insulinemic response than a lower dose does. The studies also demonstrated that oligomalt is associated with lower glycemic variability and a lower potential for inducing reactive hypoglycemia. Furthermore, the study in HV confirmed that oligomalt is fully digestible.

These data are interesting from several angles. Previously, studies in HV demonstrated that 33 g of oligomalt relative to 33 g maltodextrin induced a lower iAUC glucose response [17], and that it is well tolerated when consumed at doses of up to 180 g per day over 7 days, in addition to regular food [18]. The key novel observation across these three populations with broad characteristics, i.e., HV, PwO, and people with T2D is that oligomalt vs. maltodextrin consistently demonstrated both a lower glycemic response and an insulin-sparing effect. The insulin-sparing effect across these populations is supportive of this being a SDC that can represent a healthier carbohydrate, given that induced hyperinsulinemia has been associated with long-term deleterious effects [27]. These observations are therefore important, given that oligomalt is proposed for use in a variety of conventional foods and beverages, as well as in foods for special dietary uses.

Interestingly, in people with relatively intact beta-cell capacity (i.e., HV and PwO), the hyperinsulinemic difference between the highest dose (50 g) of maltodextrin and oligmalt was larger than with the lower dose (33 g), which in turn led to a numerically lower glucose difference, as illustrated by the slightly numerically lower glycemic iAUC in the HV in this study when consuming 50 g, where it was −15% (over 0–3 h), unlike in a previous study where it was −33% (over 0–3 h) in HV with 33 g [17], and the numerically higher iAUC (0–3 h) difference of −30% for the 33 g dose in this study vs. −15% for the 50 g dose in PwO. This dose-dependent phenomenon, i.e., a stronger incremental insulin response than glucose response, with increasing doses of rapidly acting carbohydrates in people with relatively intact physiology has also previously been observed [28] and has been ascribed in HV to mechanisms like reduced clearance of endogenous insulin with increasing glucose load of rapidly available carbohydrates [29,30,31], or induction of acute insulin resistance [30]. One potential longer term beneficial implication of this observation associated with oligmalt, relative to a rapidly acting carbohydrate, could be the improvements to insulin sensitivity by reducing the demand for insulin production from the pancreas, thereby improving fasting glycemia [32], or the post-prandial lipid profile [33], or the insulin requirement in patient populations on glucose-lowering medications. However, this remains to be demonstrated and was not assessed in these studies.

The blood kinetics of the incretin hormones GLP-1 and PYY observed in PwO and in people with T2D support the view that the hydrolysis of oligomalt is slower than maltodextrin. Glucose is a strong stimulator of incretin responses [34,35] and, as observed, a rapidly digested carbohydrate such as maltodextrin has a higher peak and a higher onset peak of the blood levels of these hormones compared to oligomalt, where we observed a later post-prandial elevation. When we contrast these observations with another study investigating the metabolic effects of another SDC, i.e., 47.5 g isomaltulose [36] vs. 47.5 g of sucrose in a comparable population (n = 10, five females, BMI 32.5 kg/m^2^, 42 years, HbA1c 37 mmol/mol), compared to our cohort of PwO with BMI ≥ 30 kg/m^2^ (n = 13, six females, BMI 32.7 kg/m2, 47 years, HbA1c 36 mmol/mol), it was observed that although isomaltulose increased GLP-1 levels overall by 170% over 4 h (i.e., a 1 h-longer observation period than in this study), the trajectory was different. In the isomaltulose study, the GLP-1 effect was greater early-on and smaller during the later period, in contrast to the present study, where we observed a significant increase in blood GLP-1 levels with oligomalt during the last 90 min. This suggests that the hydrolysis of oligomalt is slower than that of isomaltulose, since the later surge in GLP-1 and PYY is related to glucose moieties predominantly being absorbed into the distal intestine where the density of L-cell- [34,35] and PYY-producing enteroendocrine cells [37] is highest. Although not measured in this study, this can have interesting physiological consequences on satiety and gastric emptying in relation to a second-meal effect [38,39]. A similar magnitude of effect on PYY has been reported to have inverse associations with hunger sensation (r = −0.409, *p* = 0.038), and a positive with satiety sensation (r = 0.414, *p* = 0.035), in a study assessing the effects of wholemeal pasta in 14 young HV males [39]. However, this is hypothetical at this stage, since another study with SDC (raw corn starch, isomaltooligosaccharide, sucromalt) in a food matrix reported that a slight increase in gastric emptying had no relevant effect on subjective appetite [40]. Hence, at this stage, it is uncertain if oligomalt may offer a potential advantage for between-meal food behavior when used in a food matrix or, as in this study, as a single-food ingredient. Whether it may have additional benefits beyond glucose- and insulin-sparing effects, e.g., to help attenuate the perceived decline in mental and physical energy and the rise in mental and physical fatigue when compared to a high GI beverage due to a steadier blood-glucose rise and a slower blood-glucose drop, also needs to be confirmed, although this was recently reported with the use of another SDC, i.e., sucromalt [41].

A final important observation in this study was the effect on breath H_2_. In a healthy human population, with bacterial colonisation located mainly in the colon, a completely absorbed carbohydrate should not lead to any H_2_ production [24,25]. The lack of effect on breath H_2_ demonstrated by oligomalt and maltodextrin indicates that there are no remaining unabsorbed carbohydrates from oligomalt, since this would be assumed and fermented rapidly by the colonic microbiota, with the generation of gas, lactate, and short-chain fatty acids, as seen with inulin [42]. Although of short duration, there were also no reports of such an adverse event, which is also supported by the longer-term seven-day study with higher doses. Although a previously published study in HV also reported that oligmalt was fully digested [17], the present study (also involving HV) included a fiber product (inulin) as a positive control, which also enabled us to derive the estimated fiber content of oligomalt [26], which was negligible (<1%), further underscoring its full digestibility.

This study has several limitations, including the acute nature of the experiment, and no chronic dosing. The latter could be argued to be of some importance over time, given that owing to the production step, small amounts of sugars will be present with oligomalt, of which leucrose, which can be hydrolyzed to sucrose and fructose, is dominant (~5% of dry matter). Of note is that leucrose is also more slowly digested than sucrose [43], and in mechanistic studies, replacing sucrose with leucrose has led to reductions in lipid content in adipocytes and the liver [44]. It is therefore conceivable that the cumulated effect of leucrose in the context of a total carbohydrate consumption is not deleterious, and potentially may provide some additive benefits.

Although we had a reasonable representation across self-reported race, the total number was relatively small, and we had a highly homogenous population in the HV cohort, which may limit generalizability. We also used 33 g or 50 g, which is realistic for use in some products, but probably on the higher end of the amount that will be used in other food matrix combinations. Furthermore, the breath hydrogen test was only done in the HV cohort (with a relevant dose also used in other studies [45]) and not with the more senior populations or with people with comorbidities, although we speculate that the observations would not differ for these population cohorts given the consistent pattern on glucose and insulin trajectories. We can also not fully discount that there could be an interaction effect of exercise (which we did not capture) or metformin in the T2D cohort on, e.g., GLP-1 or PYY [46], which could influence the results. We do not think the small difference in baseline levels of glucose at t = 0 in the T2D cohort of 15 mg/dL (0.8 mmol/L) influenced the interpretation of the results—if anything it may have inflated the comparator results more favorably. Finally, we also did not apply gold standard techniques to assess insulin secretion or sensitivity, although single-dose experiments are not ideal for such assessments of ingredients.

## 5. Conclusions

These data confirm the glucometabolic advantages of oligomalt over maltodextrin on glucose and insulin secretion, glucose stability, and glucose variability, and they underscore its full digestibility. Oligomalt triggers a lower glycemic response and has an insulin-sparing effect, thereby highlighting its potential for being a healthier carbohydrate across a broad range of consumer characteristics.

## Figures and Tables

**Table 1:** Baseline characteristics of the different study cohorts consuming. n (%) or mean (SD).

	Healthy Volunteers	People with Overweight or Obesity	People with Type 2 Diabetes
n	15	26	22
Sex (female/male)	8 (53%)/7 (47%)	10 (39%)/16 (61%)	13 (59%)/9 (41%)
Age (years)	31 (6.8)	44 (12.3)	61 (8.9)
Race °			
Asian	0 (0%)	1 (4%)	5 (23%)
Black or African-American	0 (0%)	9 (35%)	7 (32%)
White	15 (100%)	14 (54%)	10 (45%)
Other/not reported	0 (0%)	2 (7%)	0 (0%)
Ethnicity			
Hispanic or Latino	0 (0%)	10 (39%)	7 (32%)
Not Hispanic or Latino	15 (100%)	16 (61%)	15 (68%)
SBP/DBP (mmHg)	Not assessed	117 (12.7)/73 (9.0)	123 (16)/74 (7)
Anthropometrics			
Height (cm)	172 (9.5)	175 (10.8)	166 (9.5)
Weight (kg)	67 (13.0)	92 (14.4)	88 (19.7)
Body Mass Index (BMI) (kg/m^2^)	22.6 (2.6)	29.9 (3.4)	31.8 (5.4)
Waist circumference (cm)	Not assessed	99.4 (10.5)	103.2 (13.0)
Laboratory parameters			
HbA1c (%)	Not assessed	5.3 (0.6)	7.4 (1.2)
HbA1c (mmol/mol)	Not assessed	34 (6.2)	57 (14.1)
Fasting plasma glucose (mmol/L)	4.8 (0.3)	4.9 (0.5)	8.8 (4.3)
Fasting plasma glucose (mg/dL)	86.5 (5.5)	89.6 (8.3)	160.1 (78.7)
Hematocrit (%)	Not assessed	43 (3.6)	43.3 (3.6)
Hemoglobin (g/dL)	Not assessed	13.7 (1.3)	14.1 (1.5)
eGFR ^1^ (ml/min/1.73 m^2^)	Not assessed	109 (31)	109 (31)
Concomittant medications			
Metformin	0 (0%)	0 (0%)	18 (82%)
ACE-inhibitors/AT-2 receptor blockers	0 (0%)	1 (4%)	10 (46%)
Lipid-lowering medications	0 (0%)	0 (0%)	10 (46%)
Beta-blockers	0 (0%)	0 (0%)	5 (23%)
Diuretics	0 (0%)	0 (0%)	4 (18%)
Calcium channel blockers	0 (0%)	0 (0%)	4 (18%)

°: as identified by participants, ^1^: estimated glomerular filtration rate (GFR) by MDRD formula. Abbreviations: SBP—systolic blood pressure, DBP—diastolic blood pressure. Values are mean (standard deviation) or number of participants (%).

**Table 2:** Maximum incremental glucose (Cmax), IQR, and glucose slope by consumption of maltodextrin or oligomalt in HV, PwO, or people with T2D.

	Incremental Cmax (mg/dL)			IQR (mg/dL)			Glucose Slope from Cmax to 3 h (mg/dL)		
	Oligomalt mean (SD)	Maltodextrin mean (SD)	Comparison OM vs. MD estimate (95% CI)	Oligomalt mean (SD)	Maltodextrin mean (SD)	Comparison MD vs. OM estimate (95% CI)	Oligomalt mean (SD)	Maltodextrin mean (SD)	Comparison MD vs. OM estimate (95% CI)
HV, 50 g	34.2 (14.4)	43.2 (16.2)	−10.8 (−19.8, −1.8) *p* = 0.033	23.1 (11.2)	31.5 (10.8)	−9.0 (−16.4, −1.8) *p* = 0.018	−36.2 (16.6)	−58.9 (23.1)	22.6 (10.3, 35.0) *p* = 0.001
PwO, 33 g	36.2 (13.3)	55.8 (22.9)	−19.6 (−29.2, −10.0) *p* = 0.0001	26.8 (13.5)	45.6 (13.4)	−18.8 (−27.4, −10.2) *p* < 0.0001	−42.8 (18.5)	−70.3 (23.7)	27.5 (16.6, 38.33) *p* < 0.0001
PwO, 50 g	43.5 (15.1)	61.8 (23.1)	−17.6 (−27.1, −8.2) *p* = 0.0004	31.8 (14.3)	51.1 (23.5)	−18.6 (−27.1, −10.1) *p* < 0.0001	−48.7 (18.1)	−75.0 (30.4)	25.6 (14.9, 36.3) *p* < 0.0001
T2D, 50 g	78.2 (23.1)	127.5 (34.6)	−49.4 (−67.8, −30.9) *p* < 0.0001	61.1 (20.7)	100.6 (27.6)	−39.7 (−54.8, −24.5) *p* < 0.0001	−63.4 (25.2)	−103.4 (43.6)	40.5 (22.1, 58.9) *p* = 0.0002

Abbreviations: HV—healthy volunteers, PwO—people with overweight or obesity, T2D—type 2 diabetes, MD—maltodextrin, OM—oligomalt, CI—confidence interval, Cmax—maximal concentration.

**Table 3:** Maximum incremental insulin (Cmax), time to maximum concentration (Tmax), and insulin index by consumption of maltodextrin or oligomalt in HV, PwO, or people with T2D.

	Incremental Cmax (uIU/mL)			Tmax (min)			Insulin Index (%)		
	Oligomalt Mean (SD)	Maltodextrin Mean (SD)	Comparison OM vs. MD Estimate (95% CI)	Oligomalt Mean (SD)	Maltodextrin Mean (SD)	Comparison MD vs. OM Estimate (95% CI)	Oligomalt Mean (SD)	Maltodextrin Mean (SD)	Comparison MD vs. OM Estimate (95% CI)
HV, 50 g	26.7 (13.7)	68.3 (11.7)	−41.5 (−49.2, −33.8), *p* < 0.0001	39 (9)	38 (11)	2 (−16, 20) *p = 0.822*	44 (14)	100 (0)	−56 (−67, −45) *p* < 0.0001
PwO, 33 g	26.8 (19.6, 36.5) *	64.6 (49.0, 85.0) *	0.41 (0.34, 0.50) * p < 0.0001	38 (15)	38 (10)	−1 (−9, 6) *p* = 0.7139	44 (16)	100 (0)	−56 (−64, −48) *p* < 0.0001
PwO, 50 g	32.0 (24.7, 41.5) *	82.9 (61.6, 111.4) *	0.39 (0.32, 0.47) * p < 0.0001	47 (15)	52 (18)	−4 (−11, 3) *p* = 0.2657	50 (24)	100 (0)	−50 (−59, −42), *p* < 0.0001
T2D, 50 g	14.8 (8.6, 25.3) *	31.5 (20.2, 49.0) *	0.46 (0.30, 0.71) * p = 0.0015	100 (46)	79 (43)	21 (−7, 49) *p* = 0.1377	63 (44)	100 (0)	−37 (−57, −17) *p* = 0.0005

Abbreviations: HV—healthy volunteers, PwO—people with overweight or obesity, T2D—type 2 diabetes, MD—maltodextrin, OM—oligomalt, CI—confidence interval, Cmax—maximal concentration. *: log-transformed and expressed as geometric mean (95% CI).

**Table 4:** Comparison of breath H_2_ iAUC between interventions for 0–3 h and 0–4 h. Data are log10 transformed effect estimates (95% CI) and *p*-values for between-group comparisons “Maltodextrin vs. Inulin”, “Oligomalt vs. Inulin”, and “Oligomalt vs. Maltodextrin”.

	Maltodextrin 50 g	Oligomalt 50 g
0–3 h, ppm × min		
Inulin 15 g	−117.0 (−153.6, −80.3), *p* < 0.0001	−112.8 (−151.8, −73.8), *p* < 0.0001
Maltodextrin 50 g		−4.2 (−41.8, 33.4), *p* = 0.818
0–4 h, ppm × min		
Inulin 15 g	−180.3 (−222.2, 138.4), *p* < 0.0001	−173.4 (−218.0, −128.8), *p* < 0.0001
Maltodextrin 50 g		−6.9 (−49.9, 36.1), *p* = 0.740

## Data Availability

Original data supporting these results are available on request to the corresponding author for reasonable purposes.

## Supplementary Materials

The following supporting information can be downloaded at: https://www.mdpi.com/article/10.3390/metabo14080410/s1, Table S1: inclusion criteria, Table S2: exclusion criteria, Figure S1: Structure of oligomalt; Figure S2: study scheme, Figure S3: consort diagram and cross-over study design, Table S3: baseline characteristics of the cohort of people with overweight or with obesity by BMI groups (<30 vs. ≥30 kg/m^2^), Figure S4: post-prandial glucose trajectories by consumption of maltodextrin or oligomalt in PwO by BMI categories and dose, Figure S5: post-prandial insulin trajectories by consumption of maltodextrin or oligomalt in PwO by BMI categories and dose, Figure S6: post-prandial GLP-1 trajectories by consumption of maltodextrin or oligomalt in PwO by BMI categories and dose, Figure S7: post-prandial PYY trajectories by consumption of maltodextrin or oligomalt in PwO by BMI categories and dose.
