# Supplementary material for: Oligomalt, a New Slowly Digestible Carbohydrate, Reduces Post-Prandial Glucose and Insulin Trajectories Compared to Maltodextrin across Different Population Characteristics: Double-Blind Randomized Controlled Trials in Healthy Individuals, People with Obesity, and People with Type 2 Diabetes
Source: Metabolites. 2024 Jul 26;14(8):410. doi: 10.3390/metabo14080410 (PMC11356256; doi:10.3390/metabo14080410)
Supplement: Supplementary file 1 [file metabolites-14-00410-s001.zip › metabolites-3080149-supplementary.pdf]

Supplementary Materials

Table S1. Inclusion criterion for the three different cohorts (healthy volunteers, people with overweight or obesity, people with type 2 diabetes mellitus) for studies investigating glucometabolic effects of oligomalt vs maltodextrin.

	**Healthy volunteers**	**People with overweight or obesity**	**People with type 2 diabetes mellitus**
**Inclusion criteria**			
Signed and understood informed consent form	√	√	√
Gender	*Both*	*Both*	*Both*
Age, years	*18-45*	*≥18*	*≥18*
BMI, kg/m^2	*18.5-29.9*	*≥ 25.0*	*Not specified*
Other	*Healthy (based on anamnesis)*	*Fasting plasma glucose ≤ 125 mg/dL*	*Established diagnosis of T2D (or HbA1c 6.5 – 10.0%)*
Other	*N/A*	*N/A*	*Treatment naïve or on metformin at a daily dose of 1000-3000 mg (stable ≥ 3 months prior to screening)*
Other	*N/A*	*N/A*	*Hematocrit ≥ 34.0% for females and 40% for males*
Other	*N/A*	*N/A*	*Hemoglobin ≥ 11.0 g/dL for females and 13.5 g/dL for males*

Table S2. Exclusion criterion for the three different cohorts (healthy volunteers, people with overweight or obesity, people with type 2 diabetes mellitus) for studies investigating glucometabolic effects of oligomalt vs maltodextrin.

	**Healthy volunteers**	**People with overweight or obesity**	**People with type 2 diabetes mellitus**
**Exclusion criteria**			
Pregnant or lactating women	√	√	√
Diabetes mellitus (DM)	*Type 1/type 2 DM*	*Type 1/type 2 DM, or 2-h plasma glucose ≥ 200 mg/dL ≤ 6 weeks prior to screening.*	*Type 1 DM*
Known food allergy/intolerance to test product	√	√	√
Major medical/ surgical event ≥3 months potentially interfering with study procedures and assessments	√	√	√
Abnormal bowel transit, history of a GI disorder (*e.g.,* inflammatory bowel disease, diverticular diseases, colon cancer), or history of chronic constipation with passage of f< 3 spontaneous bowel movements per week or chronic/recurrent diarrhea with spontaneous bowel movements > 3 times/day	√	√	√
Concomitant medication potentially interfering with study procedures and assessment (e.g., antibiotics, medications impacting transit time, colonoscopy, irrigoscopy, bowel cleansing procedures < 4 weeks) prior to dosing	√	√	√
Recent episode of an acute gastrointestinal illness	√	√	√
Alcohol intake > 2 servings/day	√	√	√
Current daily cigarette smoking	√	√	√
Unable to comply with the protocol	√	√	√
Family or hierarchical relationships with Clinical Innovation Lab team.	√	N/A	N/A
Glucose lowering medication	N/A	Any	Any except metformin
Current use of weight loss interventions or treatment with anorectic drugs.	N/A	√	√
Current treatment with systemic steroids (inhaled or topical steroids is permitted).	N/A	√	√
Participation in another clinical study with any investigational drug/new chemical entity ≤30 days or 5 $t_{½}$ (whichever is longer) prior to screening.	N/A	√	√

Current treatment with anticoagulants or antithrombotic agents (warfarin, NOACs, heparin, platelet inhibitors).	N/A	√	√
Donation of blood, or significant amount of blood loss ≤8 weeks prior to screening. Participants must also agree to not donate blood within 8 weeks after their last visit.	N/A	√	√

Figure S1 Structure of oligomalt. Figure from Lamothe, L.M.; Francey, C.; Lerea-Antes, J.s.; Rytz, A.; D´Urzo, C.;Delodder, F.;Piccardi, N.; Curti, D.; Muricano Martinez, P.; Darimont, C.; Vafiadi, C. Effects of α-D-glucans with alternating 1,3/1,6 α-D-glucopyranosyl linkages on postprandial glycemic response in healthy subjects. Carbohydrate Polymer Technologies and Applications 2022, 4, 100256. https://www.sciencedirect.com/science/article/pii/S2666893922000718

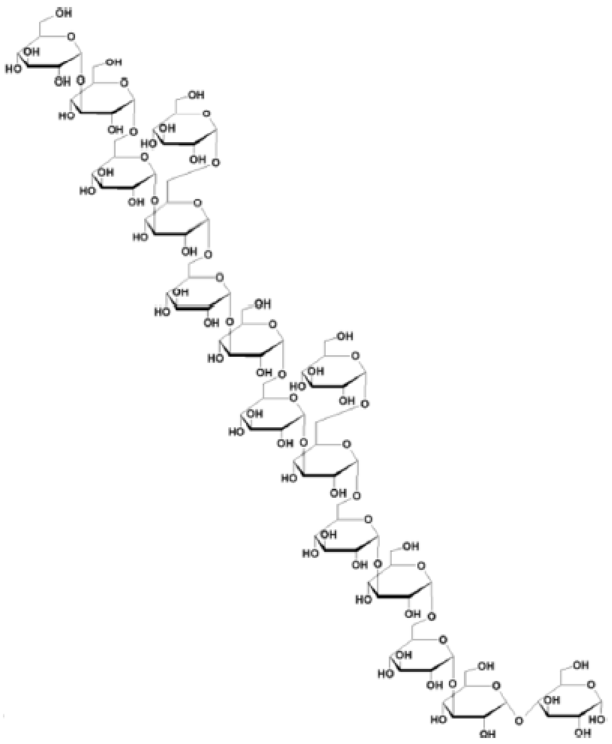

Creative Commons Attribution-NonCommercial-NoDerivs 4.0 International License (CC BY-NC-ND 4.0)

Figure S2. Study scheme

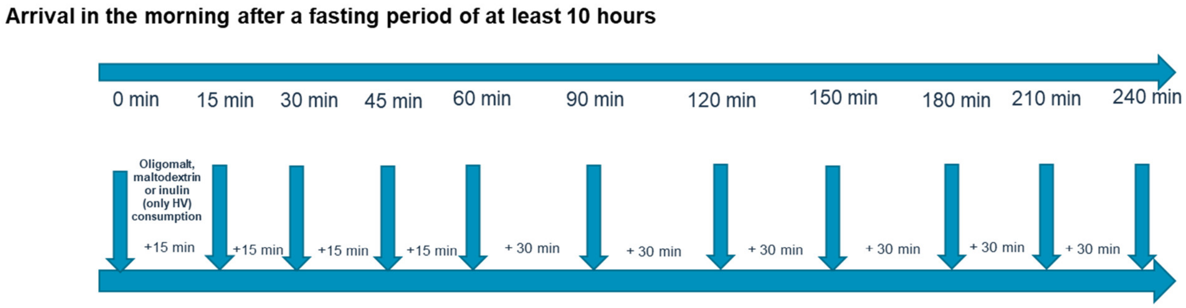

T	0	15	30	45	60	90	120	150	180	210	240
HV	Glucose Insulin, H2 breath test	Glucose Insulin, H2 breath test	Glucose Insulin, H2 breath test	Glucose Insulin, H2 breath test	Glucose Insulin, H2 breath test	Glucose Insulin, H2 breath test	Glucose Insulin, H2 breath test	Glucose Insulin, H2 breath test	Glucose Insulin, H2 breath test	H2 breath test	H2 breath test
PwO	Glucose, insulin, GLP-1, PYY	Glucose, insulin, GLP-1, PYY	Glucose, insulin, GLP-1, PYY	Glucose, insulin, GLP-1, PYY	Glucose, insulin, GLP-1, PYY	Glucose, insulin, GLP-1, PYY	Glucose, insulin, GLP-1, PYY	Glucose, insulin, GLP-1, PYY	Glucose, insulin, GLP-1, PYY	No test/ Sampling	No test/ sampling
T2D	Glucose, insulin, GLP-1, PYY	Glucose, insulin, GLP-1, PYY	Glucose, insulin, GLP-1, PYY	Glucose, insulin, GLP-1, PYY	Glucose, insulin, GLP-1, PYY	Glucose, insulin, GLP-1, PYY	Glucose, insulin, GLP-1, PYY	Glucose, insulin, GLP-1, PYY	Glucose, insulin, GLP-1, PYY	No test/ Sampling	No test/ Sampling

Abbreviations: HV – healthy volunteers, PwO – people with obesity, T2D – type 2 diabetes mellitus, H2 - hydrogen

Figure S3. Consort diagram and cross over study design.

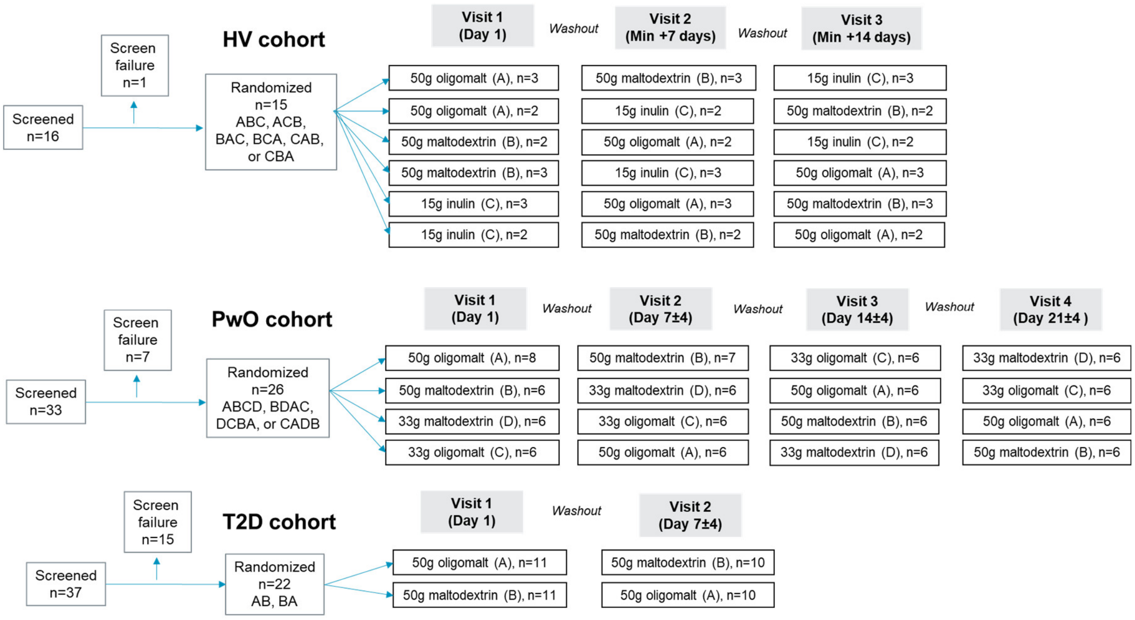

Abbreviations: HV – healthy volunteers, PwO – people with obesity, T2D – type 2 diabetes mellitus. As shown, in the HV cohort, there was one screen failure, and all 15 randomized participants completed their allocated sequence and test. In the PwO cohort, there were 7 screen failures, and out of 26 randomized participants, 24 completed their allocated sequence and test (one participant withdrew consent after the first period, and one after the second period). In the T2D cohort, there were 15 screen failures, and out of the 22 randomized participants, 20 completed their allocated sequence and test (two participants withdrew their consent after first period).

Table S3. Baseline characteristics of the cohort of people with overweight or obesity (PwO) by BMI groups (< 30 vs ≥ 30 kg/m^2). n (%), or mean (SD).

	PwO		
	BMI ≥ 25.0 kg/m^2 *n=26*	BMI 25.0-29.9 kg/m^2 *n=13*	BMI ≥ 30 kg/m^2 *n=13*
Sex (female/male)	10 (39%)/16 (61%)	4 (31%)/9 (69%)	6 (46%)/7 (54%)
Age (years)	44 (12.3)	40 (12.1)	47 (11.9)
Race°			
Asian	1 (4%)	1 (8%)	0 (0%)
Black or African American	9 (35%)	3 (23%)	6 (46%)
White	14 (54%)	8 (61%)	6 (46%)
Other/not reported	2 (7%)	1 (8%)	1 (8%)
Ethnicity			
Hispanic or Latino	10 (39%)	5 (39%)	5 (39%)
Not hispanic or Latino	16 (61%)	8 (61%)	8 (61%)
Weight (kg)	92 (14.4)	85 (13.7)	98 (12.0)
Body Mass Index (BMI) (kg/m^2)	29.9 (3.4)	27.2 (1.5)	32.7 (2.3)
Waist circumference (cm)	99.4 (10.5)	95.0 (8.0)	103.7 (11.0)
SBP/DBP (mmHg)	117 (12.7)/73 (9.0)	117 (10.1)/72 (5.0)	117 (15.4)/75 (11.9)
Laboratory parameters			
HbA1c (%)	5.3 (0.6)	5.1 (0.7)	5.4 (0.4)
HbA1c (mmol/mol)	34 (6.2)	33 (7.8)	36 (5.2)
Fasting plasma glucose (mmol/L)	4.9 (0.5)	5.1 (0.5)	4.9 (0.4)
Fasting plasma glucose (mg/dL)	89.6 (8.3)	91.8 (9.0)	87.4 (7.1)
Hematocrit (%)	43 (3.6)	43.5 (4.1)	41.7 (3.0)
Hemoglobin (g/dL)	13.7 (1.3)	14.0 (1.4)	13.4 (1.1)
eGFR[1] (ml/min/1.73m^2)	109 (31)	102 (28)	116 (33)

°: as identified by participants,[1]: estimated glomerular filtration rate (GFR) by MDRD formula. Abbreviations: SBP – systolic blood pressure, DBP – diastolic blood pressure

Figure S4. Postprandial glucose trajectories by consumption of maltodextrin or oligomalt in PwO by BMI categories and dose; a) BMI < 30 kg/m^2 33 g oligomalt/maltodextrin, b) BMI < 30 kg/m^2 50 g oligomalt/maltodextrin, c) BMI ≥ 30 kg/m^2 33 g oligomalt/maltodextrin, d) BMI ≥ 30 kg/m^2 50 g oligomalt/maltodextrin

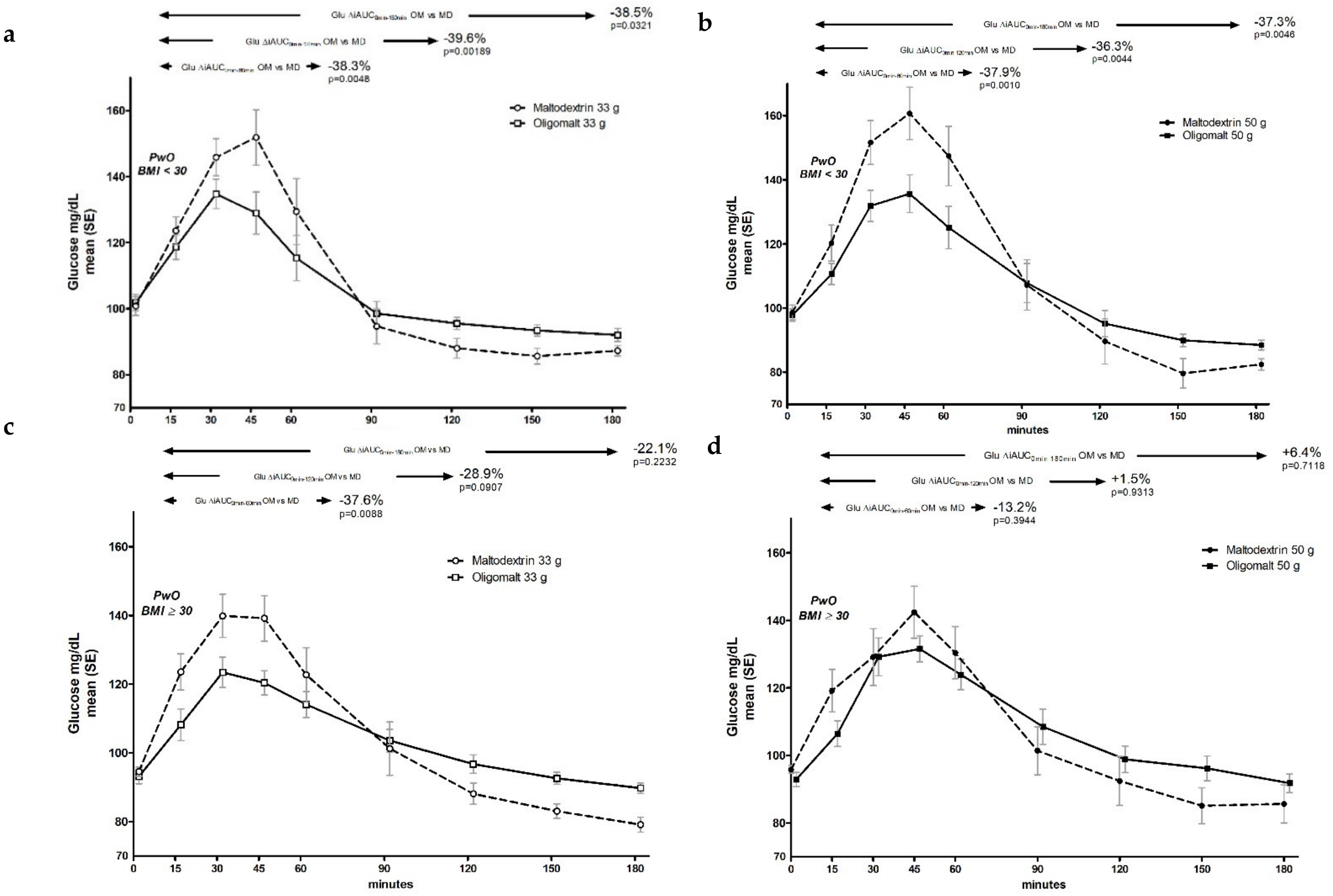

Abbreviations: PwO – people with obesity, MD – maltodextrin, OM - oligomalt

Figure S5. Postprandial insulin trajectories by consumption of maltodextrin or oligomalt in PwO by BMI categories and dose; a) BMI < 30 kg/m² 33 g oligomalt/maltodextrin, b) BMI < 30 kg/m² 50 g oligomalt/maltodextrin, c) BMI ≥ 30 kg/m² 33 g oligomalt/maltodextrin, d) BMI ≥ 30 kg/m² 50 g oligomalt/maltodextrin

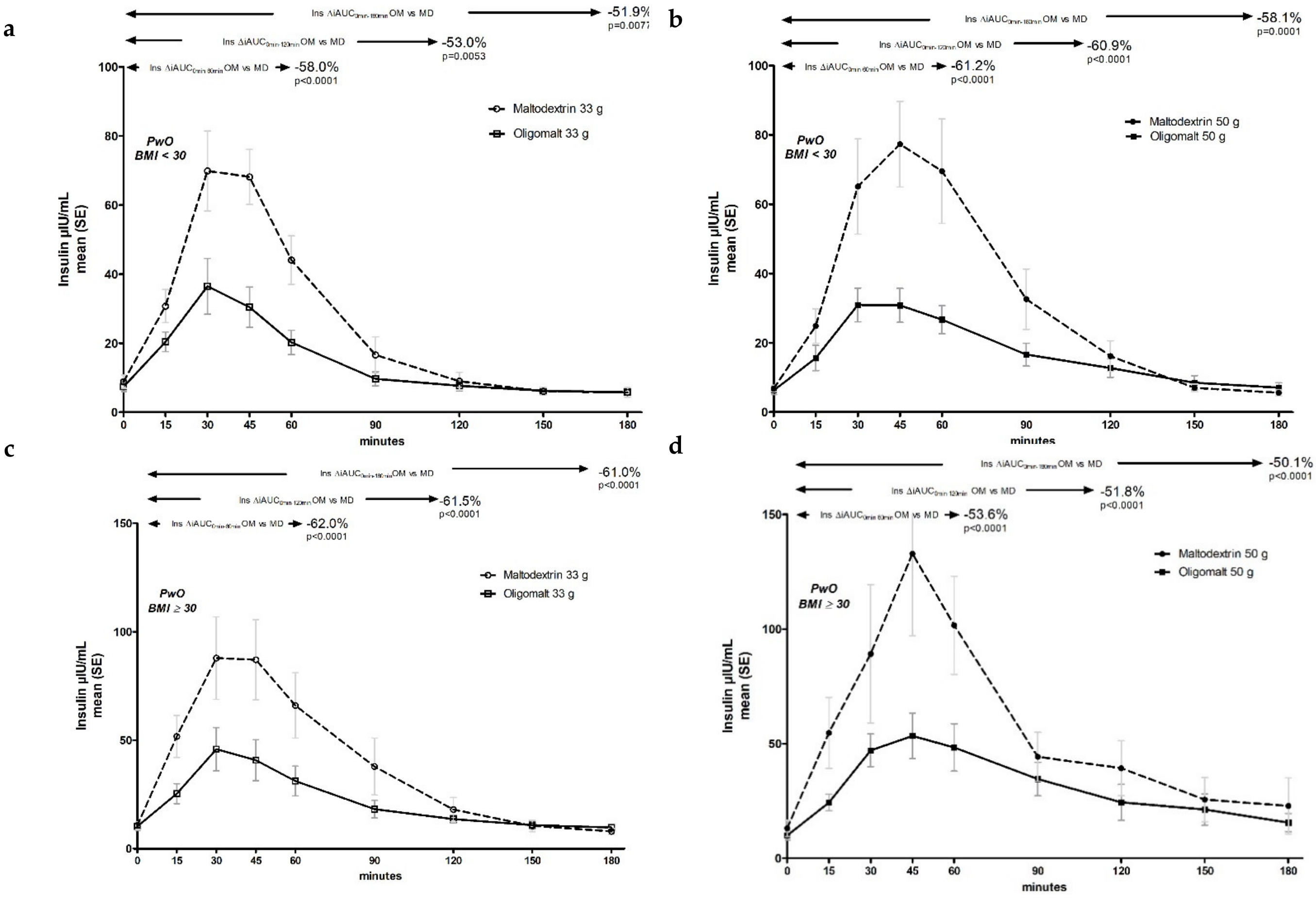

Abbreviations: PwO – people with obesity, MD – maltodextrin, OM - oligomalt

Figure S6. Postprandial GLP-1 trajectories by consumption of maltodextrin or oligomalt in PwO by BMI categories and dose; a) BMI < 30 kg/m² 33 g oligomalt/maltodextrin, b) BMI < 30 kg/m² 50 g oligomalt/maltodextrin, c) BMI ≥ 30 kg/m² 33 g oligomalt/maltodextrin, d) BMI ≥ 30 kg/m² 50 g oligomalt/maltodextrin

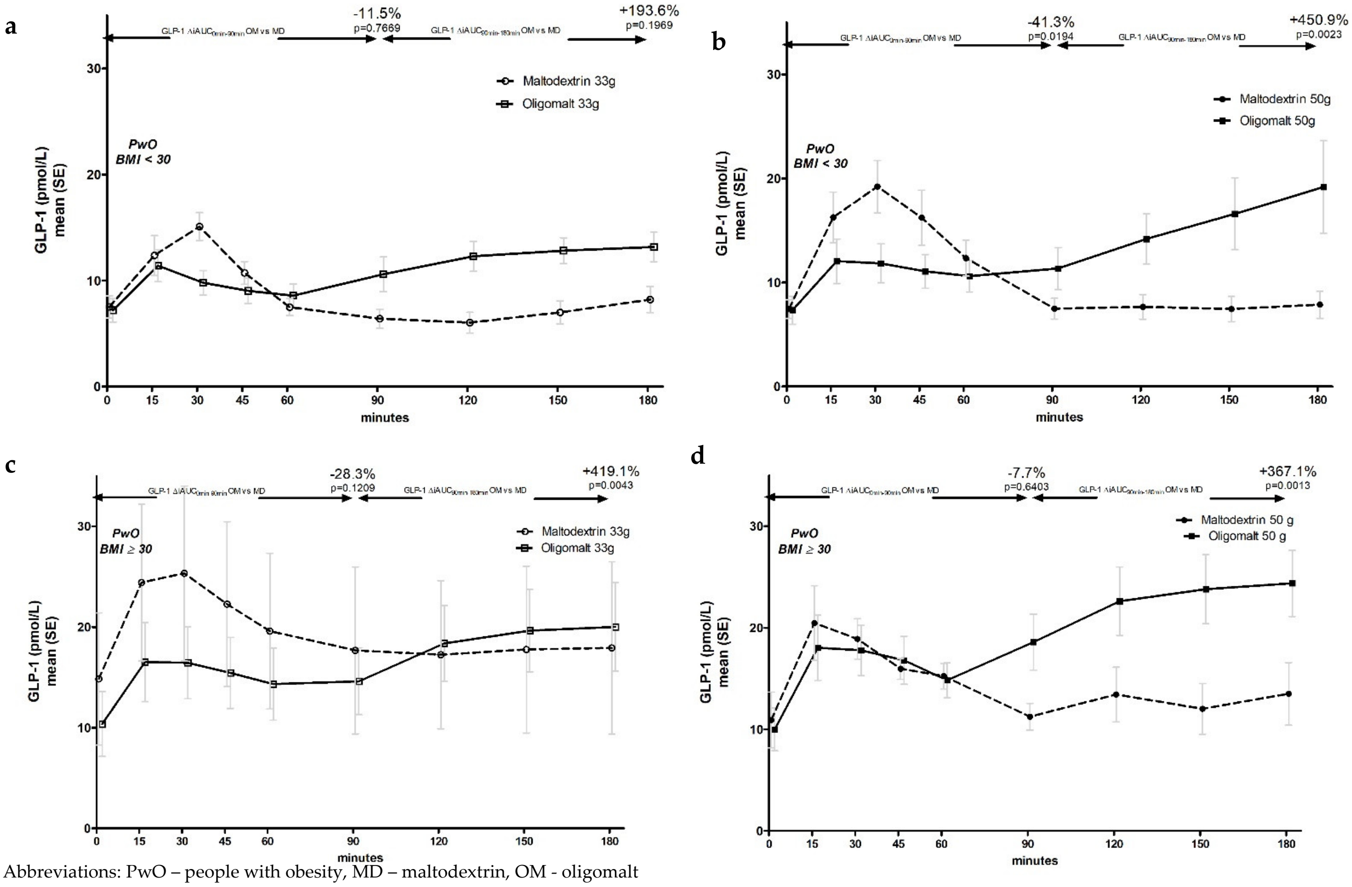

Abbreviations: PwO – people with obesity, MD – maltodextrin, OM - oligomalt

Figure S7. Postprandial PYY trajectories by consumption of maltodextrin or oligomalt in PwO by BMI categories and dose; a) BMI < 30 kg/m² 33 g oligomalt/maltodextrin, b) BMI < 30 kg/m² 50 g oligomalt/maltodextrin, c) BMI ≥ 30 kg/m² 33 g oligomalt/maltodextrin, d) BMI ≥ 30 kg/m² 50 g oligomalt/maltodextrin

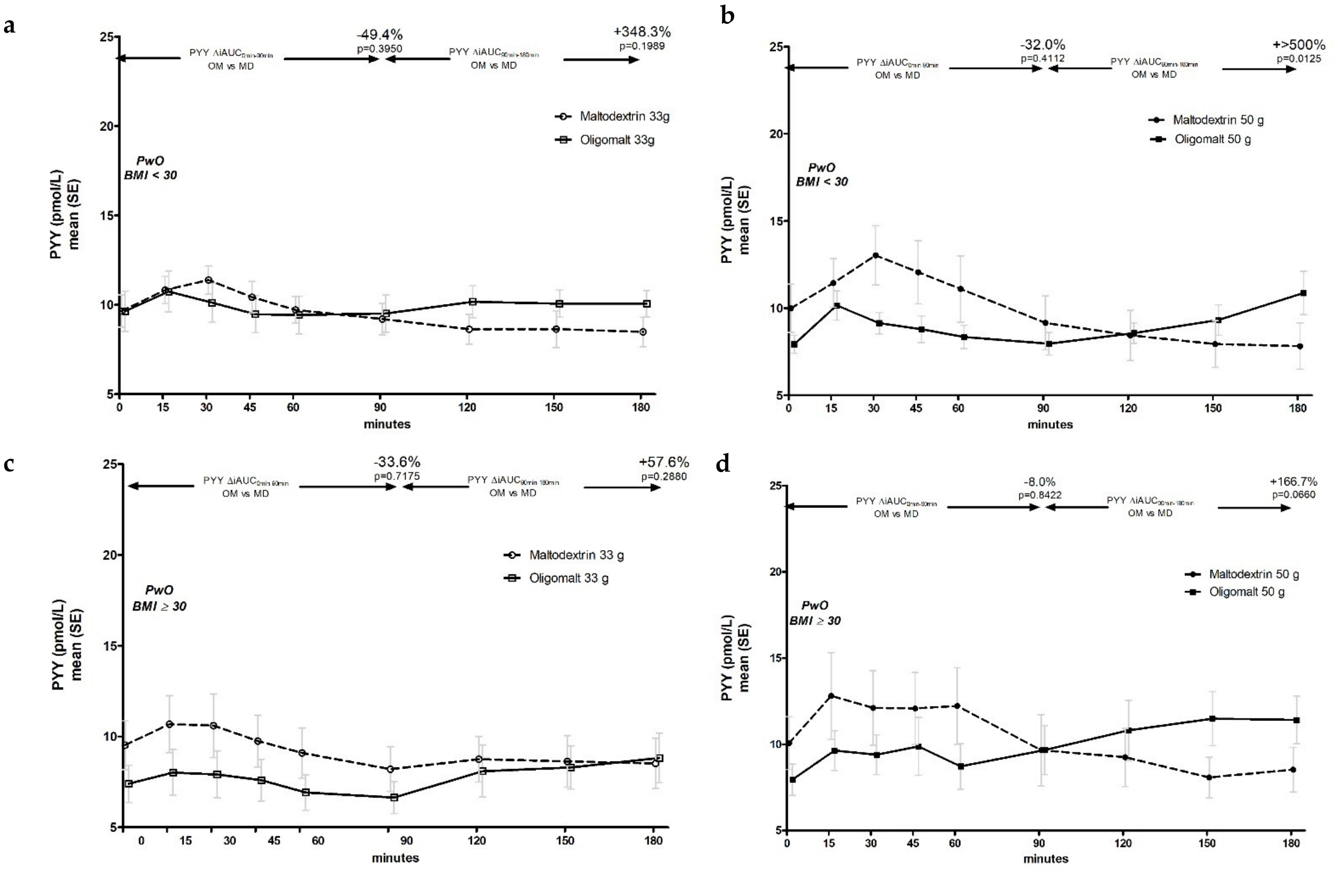

Abbreviations: PwO – people with obesity, MD – maltodextrin, OM - oligomalt
